# Anatomical relationship between the omohyoid muscle and the internal jugular vein on ultrasound guidance

**DOI:** 10.1186/s12871-022-01723-4

**Published:** 2022-06-13

**Authors:** Yun Yang, Xinqiang Wang, Weiliang Mao, Tongyun He, Zhaodong Xiong

**Affiliations:** Department of Anesthesiology, the First People’s Hospital of Huzhou, the Affiliated Hospital of Huzhou Teachers College, Guangchanghou Road 158th, 313000 Huzhou, People’s Republic of China

**Keywords:** Ultrasound, Omohyoid muscle, Internal jugular vein

## Abstract

**Background:**

Internal jugular vein catheterization is widely used in clinical practice, and there are many related studies on internal jugular vein catheterization. However, the omohyoid muscle, which is adjacent to the internal jugular vein, is a rarely mentioned muscle of the infrahyoid muscles group. The purpose of this study is to explore the anatomical relationship between the omohyoid muscle and the internal jugular vein on ultrasound guidance and provide a theoretical reference for jugular puncture and catheterization.

**Methods:**

The study included 30 volunteers. The volunteer’s head lay in the neutral position and was then turned to the left at an angle of 30°, 45° and 60° with the bed surface, as verified using an adjustable protractor. A high-frequency ultrasound probe (6–14 Hz) was used to examine the plane of the apex of sternocleidomastoid triangle (PAST), the triangle consists of anatomical landmarks: a base was clavicle, its sides – heads of sternocleidomastoid muscle. And the plane of the middle of sternocleidomastoid triangle(PMST) which was a horizontal line, connecting midpoints of both sides. The right omohyoid muscle (OM) and the right internal jugular vein (IJV) were observed and recorded for statistical analysis.

**Results:**

There were statistically significant differences in the number of overlapping cases of OM and IJV at each head rotation angle between the PAST and PMST groups. There were statistically significant differences between the angles which OM and IJV centre point line and the left horizontal position of the PAST and PMST at different body angles.

**Conclusion:**

The traditional middle route puncture point is the apex of the sternocleidomastoid triangle, which can effectively avoid injury to the omohyoid muscle, to an extent.

**Trail registration:**

ChiCTR2000034233, Registered 29/06/2020. www. Chinese Clinical Trial Registry.gov.

## Background

The placement of a central venous access into the internal jugular vein(IJV) is a commonly practiced clinical technique that allows fluid and blood transfusion and other rescue treatments. It is widely used to treat critically ill patients with severe trauma, shock, and acute circulatory failure. It can also be used for total parenteral nutrition therapy, long-term intravenous fluid and antibiotic therapy, and temporary cardiac pacemaker transcatheter installation[1]. The reported incidence of mechanical complications following central venous catheterization is between 1.1% and 17%, and depending on its definition, the most common complication is carotid artery injury[2]. Therefore, many studies have been conducted on the relationship between the IJV and carotid artery, as well as ultrasonic observation of the effect of positive pressure ventilation and body position on the size of the IJV[3, 4] and relevant studies on the catheterization of the IJV[5]. However, few people are concerned about the muscles that surround the IJV, and few people are careful not to damage them during the puncture, such as the omohyoid muscle(OM). The OM has an inferior belly originating from the superior border of the scapula, near the suprascapular notch. This muscle then passes deep to the sternocleidomastoid where its superior belly passes almost vertically upward next to the lateral border of sternohyoid to attach to the inferior border of the body of the hyoid bone lateral to the insertion of sternohyoid[6]. OM can ensure the smooth completion of swallowing. And the upper and lower abdomen of the OM can continuously improve the neck pressure and effectively relieve the compression of the carotid artery by the OM during swallowing. Lesions of the OM are characterized by discomfort or pressure in the patient during swallowing, accompanied by a raised mass on the affected side[7]. Although we found a close relationship between the OM and the IJV[8], clinicians often overlook the relationship in clinical practice. The purpose of this study was to explore the anatomical relationship between the OM and the IJV under ultrasound to provide a theoretical reference for internal jugular puncture and catheterization.

## Methods

This study was approved by the Ethics Committee of Medical Research and Clinical Trials of Huzhou First People's Hospital. All methods were carried out in accordance with relevant guidelines and regulations and Declaration of Helsinki. This clinical trial was registered at http://www.chictr.org.cn (No. ChiCTR2000034233, Registered 29/06/2020). Thirty volunteers (15 women and 15 men), who were healthy, were included from September 2020 to June 2021. Participant age ranged from 18 to 80 years. In addition, included patients must have the ability to cooperate with the observation throughout the process. Volunteers were excluded if they had thyroid disease, abnormal neck anatomy, history of neck puncture or surgery. Volunteers who could not correctly communicate were also excluded.

**Fig. 1:**
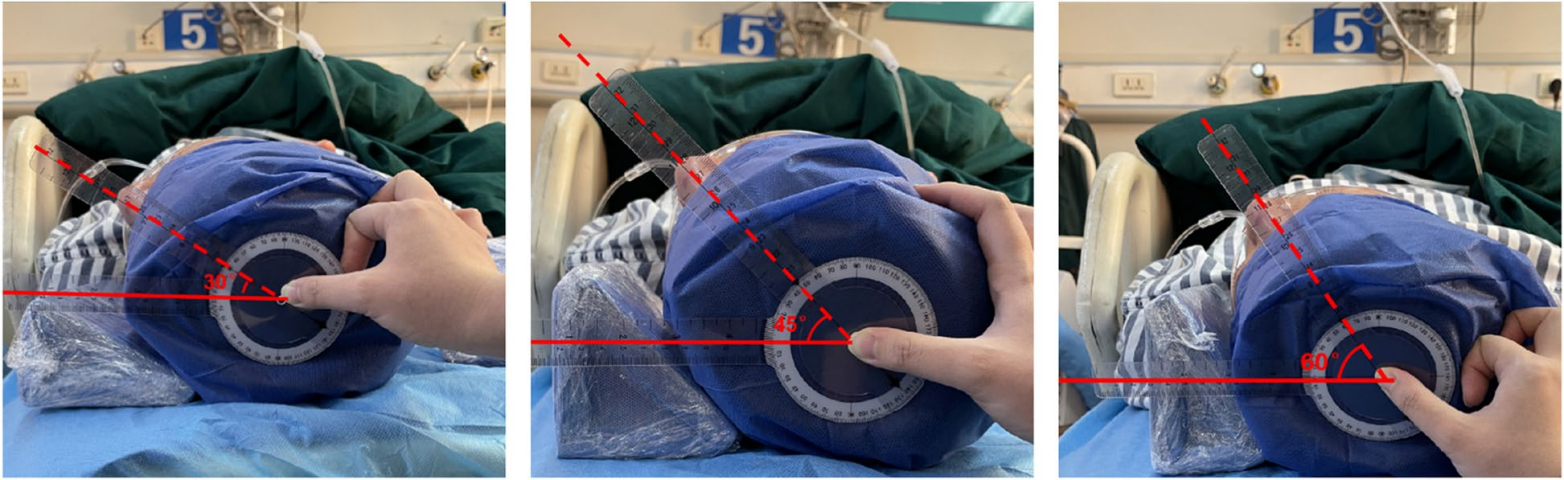
Each subject’s head was rotated 30°,45°,60° left as verified using the Custom Angular Model

**Fig. 2:**
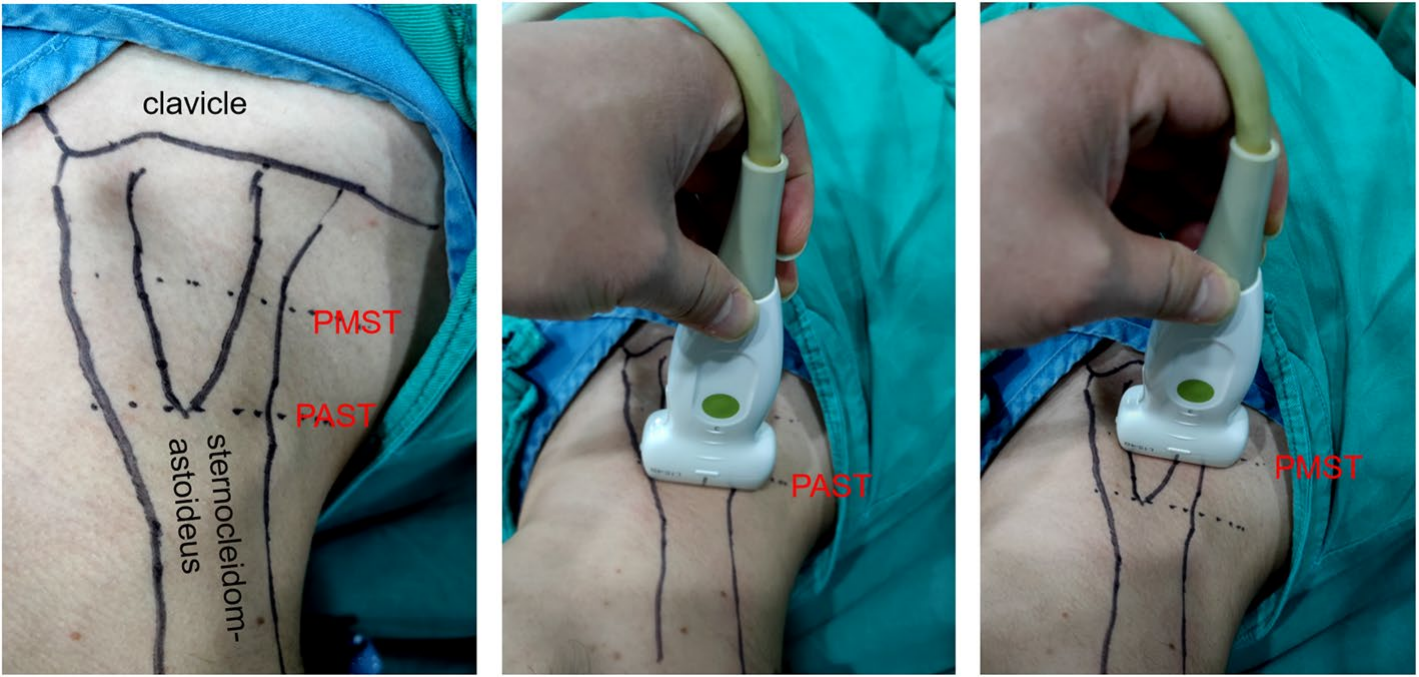
Place the probe in the horizontal body position on the right PAST and PMST planes

Each volunteer’s head lay in the neutral position and was then turned to the left at an anlge of 30°, 45° and 60° with the bed surface, as verified using an adjustable protractor (Fig. 1).An ultrasonographic study was performed using the Wisonic Navi Ultrasound System (Shenzhen Wisonic Medical Technology Co., Ltd. ShenZhen, China) using a linear (6–14 MHz) probe.At each of the above angles (Fig. 2). The probe was placed in the horizontal position on the plane of the apex of sternocleidomastoid triangle (PAST), which is formed the triangle consists of anatomical landmarks: a base was clavicle, its sides – heads of sternocleidomastoid muscle. And the plane of the middle of sternocleidomastoid triangle(PMST) which was a horizontal line, connecting midpoints of both sides. Ultrasound was applied gently to avoid distorting the underlying low-pressure venous structures. The relevant indicators of the right OM and IJV were observed, measured and recorded; then the data were compared to determine whether there were differences between the different head angles in each plane. Additionally, we performed a comparison to determine whether there were differences in the data between the same head angle of PAST and PMST planes.

**Fig. 3:**
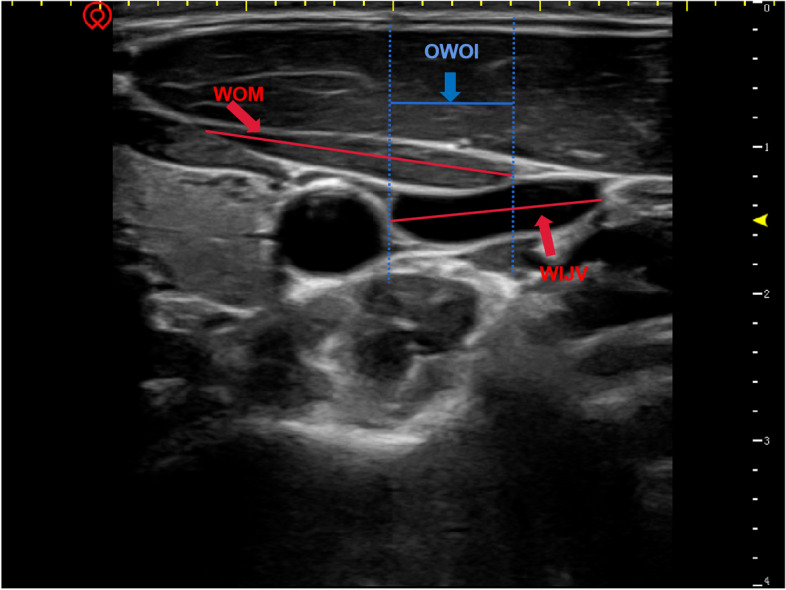
WOM: the width of omohyoid muscle; WIJV: the width of internal jugular vein (right side)

**Fig. 4:**
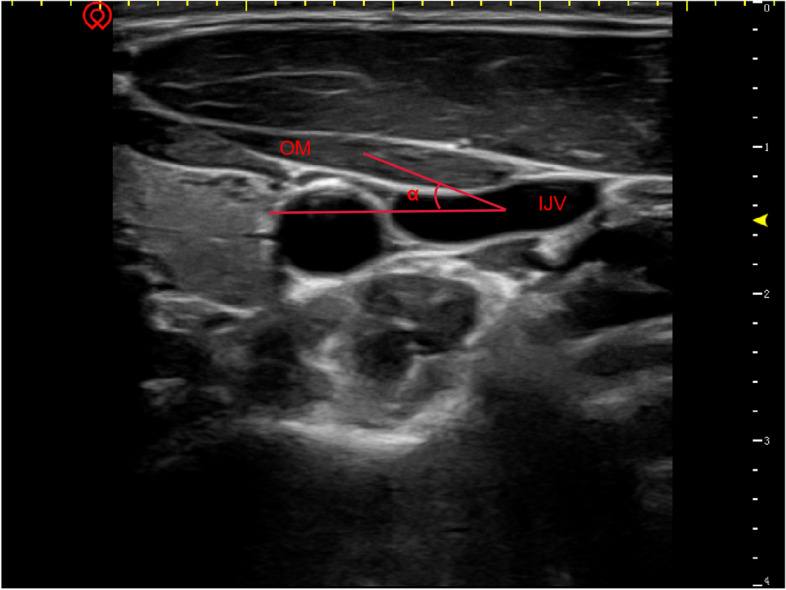
OM: omohyoid muscle; IJV: internal jugular vein; α: The angle formed by the line connecting the centre point of OM and the IJV and the horizontal plane on the left side (right side)

The data collected included the following: width of the omohyoid muscle (WOM, cm), the width of the internal jugular vein (WIJV, cm) (Fig. 3), the angle α formed by the line connecting both the centre point of the OM and the IJV, with the left horizontal plane (Fig. 4), the overlapping width of the omohyoid muscle and internal jugular vein (OWOI, cm) in the two planes (Fig. 3), the number of overlapping cases, and the overlapping rate (OR) was defined as ratio of overlapping width of omohyoid muscle and internal jugular vein to width of internal jugular vein, OR = [OWOI/WIJV] × 100%.

All of the data are presented as the mean ± standard deviation (SD), median (interquartile range), or number (%), as appropriate. The measurement data were analysed by Shapiro–Wilk. Normally distributed data that satisfied the homogeneity test of variance were tested by ANOVA, if not by Kruskal–Wallis. The abnormally distributed data were analysed by Kruskal–Wallis to compare among groups. Pearson’s chi-squared test was used to assess the comparison among groups in count data. Data were analysed using SPSS software (SPSS 22.0, SPSS, Inc., Chicago, IL, USA). A p value of less than 0.05 was considered to be a significant difference.

## Results

**Fig. 5:**
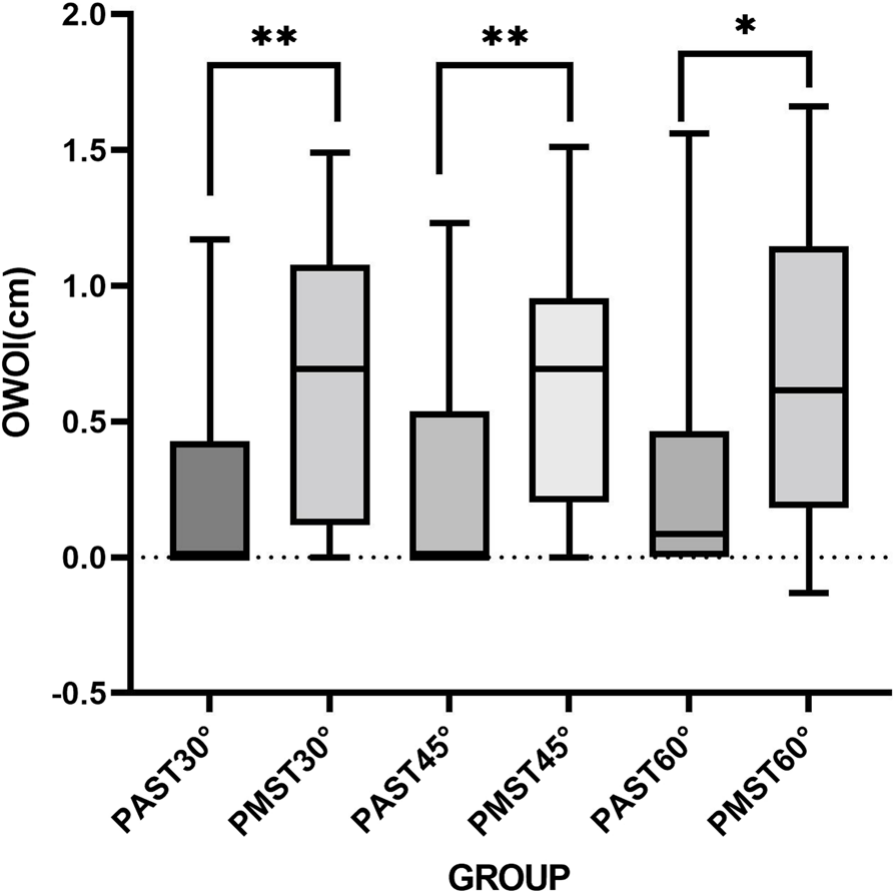
The OWOI of the PAST plane and the PMST plane. *P < 0.05, **P < 0.001

**Fig. 6:**
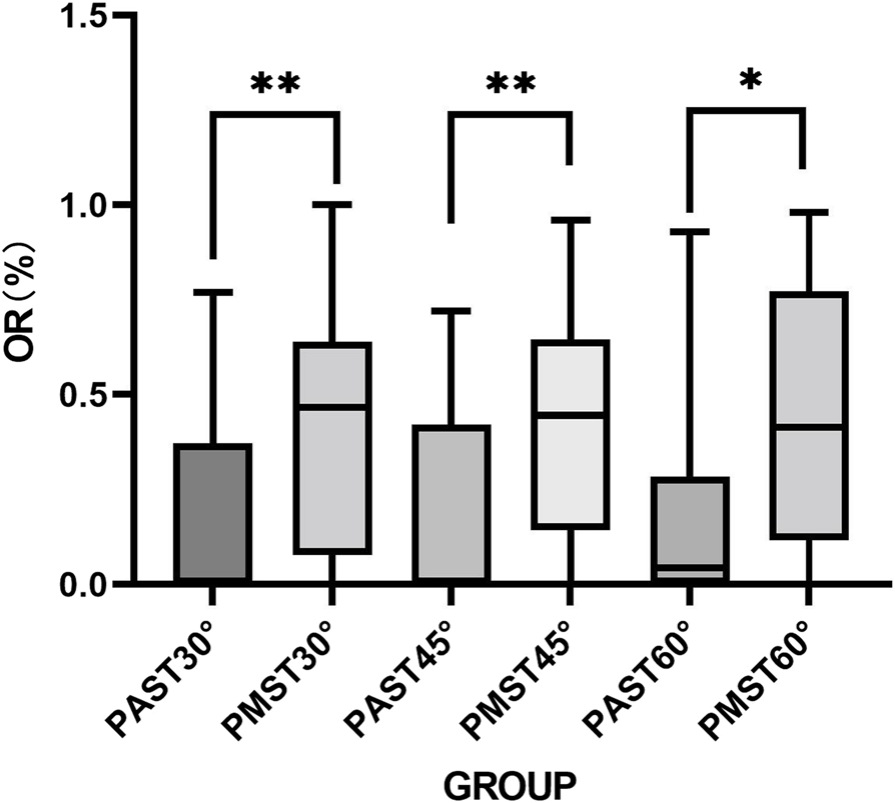
The OR of the PAST plane and the PMST plane. *P < 0.05, **P < 0.001

**Fig. 7:**
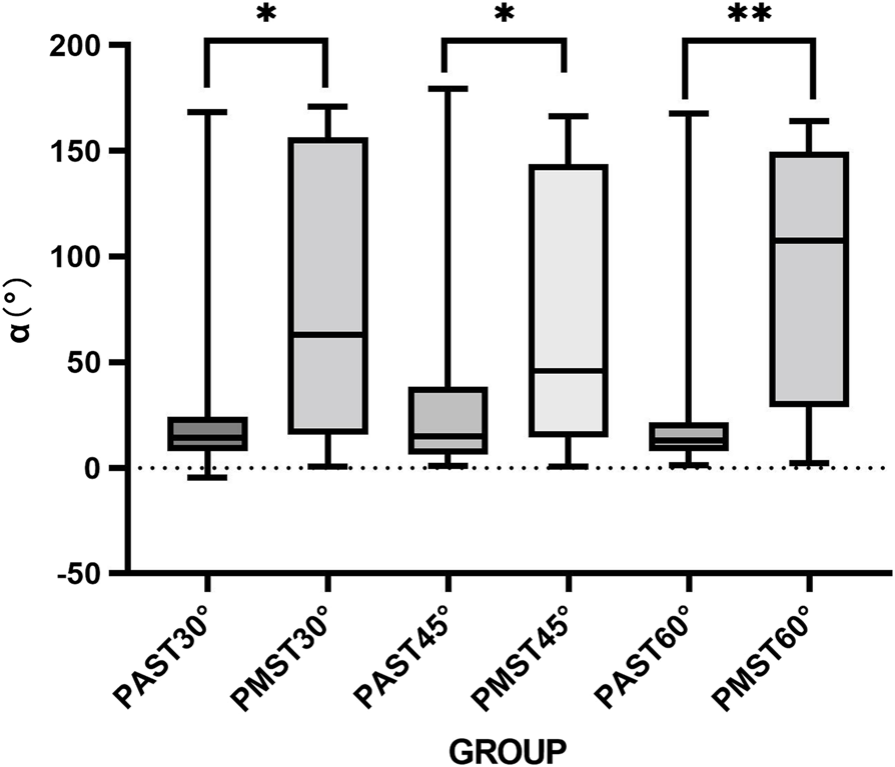
The α of the PAST plane and the PMST plane. *P < 0.05, **P < 0.001

There were 30 participants, including 15 males and 15 females, aged 39.70 ± 17.74 years, with a height of 166.03 ± 9.11 cm and weight 61.35 ± 11.64 kg. The WIJV of the PAST plane (1.35 ± 0.40 cm) was significantly lower than that of the PMST plane (1.56 ± 0.36 cm) at 30 degree angles (*P* < 0.05) (see Table 1). The number of cases where OM and IJV overlap in the PAST plane at each angle is significantly less than that in the PMST plane (*p* < 0.05), and there is no significant difference in the number of cases where OM and IJV overlap at different angles in the same plane (see Table 2). At the same angle, the OWOI and OR of the PAST plane were significantly lower than those of the PMST plane (*p* < 0.05) (see Fig. 5 and Fig. 6). In addition, by comparing the α angles of PAST and PMST, it was found that the α angle of PAST was significantly smaller than that of PMST at the same head angle (*p* < 0.05) (see Fig. 7).

**Table 1:** WOM and WIJV Comparison of PAST and PMST (${x}_{\pm s}$, cm)

	**PAST**			**PMST**		
	30°	45°	60°	30°	45°	60°
WOM(cm)	1.32 ± 0.42	1.41 ± 0.44	1.35 ± 0.45	1.34 ± 0.50	1.27 ± 0.53	1.36 ± 0.54
WIJV(cm)	1.35 ± 0.40^*^	1.39 ± 0.36	1.42 ± 0.38	1.56 ± 0.36^*^	1.54 ± 0.35	1.50 ± 0.41

^*^: PAST Compared with PMST, *p* < 0.05

**Table 2:** The positional relationship between OM and IJV in two planes of PAST and PMST (case)

	**PAST**			**PMST**		
	30°	45°	60°	30°	45°	60°
OM and IJV overlap cases	11	13	16	5^*^	25^*^	26^*^
OM and IJV do not overlap case	19	17	14	5	5	4
Total	30	30	30	30	30	30

^*^: PAST Compared with PMST, *p* < 0.05

## Discussion

Our study mainly observed the anatomical relationship between the OM and the IJV in two planes under ultrasound guidance. The selection of the two planes corresponds to the punctured plane of the traditional middle method and posterior method for IJV puncture. The main results included the overlap of the IJV and OM at the corresponding head angle for each plane and the anatomical angle α of the IJV under ultrasound with OM. We found that the lower the punctured plane was, the more significant the overlap and α of the IJV and OM.

The overlapping relationship between OM and IJV has not been previously documented. Our results showed that there was no significant difference in the overlap of the IJV and OM at different head angles on the same plane. Then, the number of overlapping cases, OWOI, and the OR of the PAST planes with the same angle are smaller than those of the PMST plane. This also indicates a greater likelihood of injury to the OM by a puncture at a relatively low level.

The results show that for the α angle in the PAST plane, the median values of the three individual bit angles were 14.17°, 14.97°, and 12.89°, indicating that the OM is mainly located on the upper medial side of the IJV in the PAST plane. The α angle in the PMST plane was larger, and the median values of the three individual bit angles were 62.75°, 45.68°, and 107.33°, indicating that the right OM was mainly located on the medial and lateral surfaces of the right IJV. The PAST plane was the insertion point for our middle puncture. Generally, the puncture needle was oriented towards the posterolateral during middle puncture, which also suggested that the probability of injury to OM during middle puncture was lower. However, the PMST plane corresponds to the posterior puncture point, and the OM below the PMST plane is mainly located on the upper and lateral sides of the IJV, and the needle insertion direction of posterior puncture is generally towards the posterior and medial sides, which also indicates that the probability of passing through the OM during puncture is higher.

**Fig. 8:**
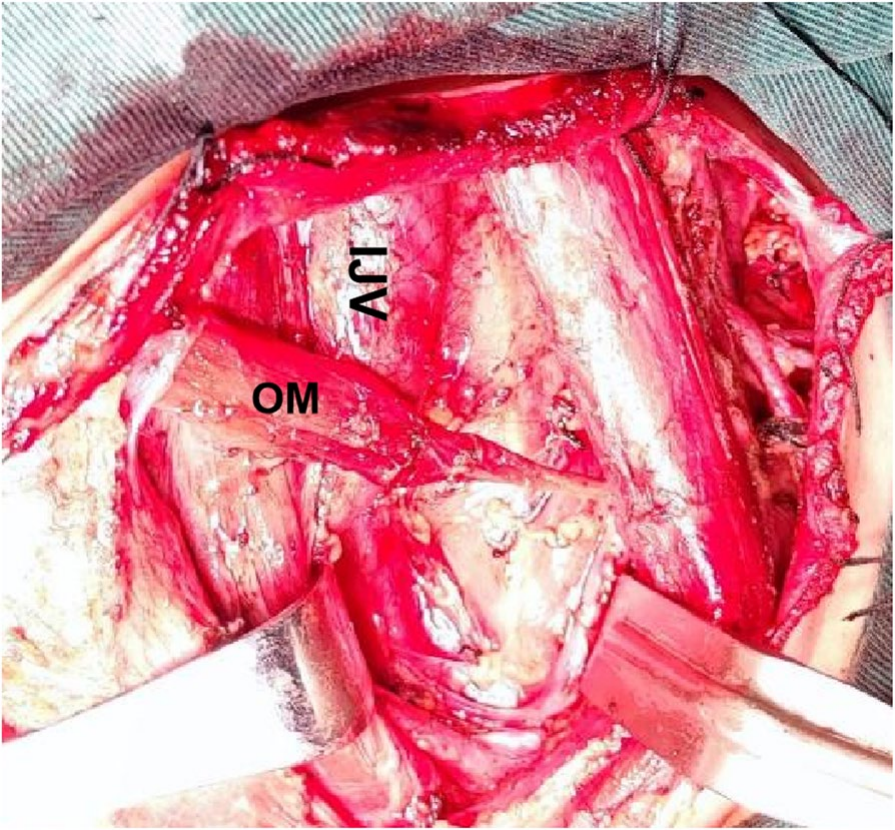
Anatomical relationship between the right OM and IJV

**Fig. 9:**
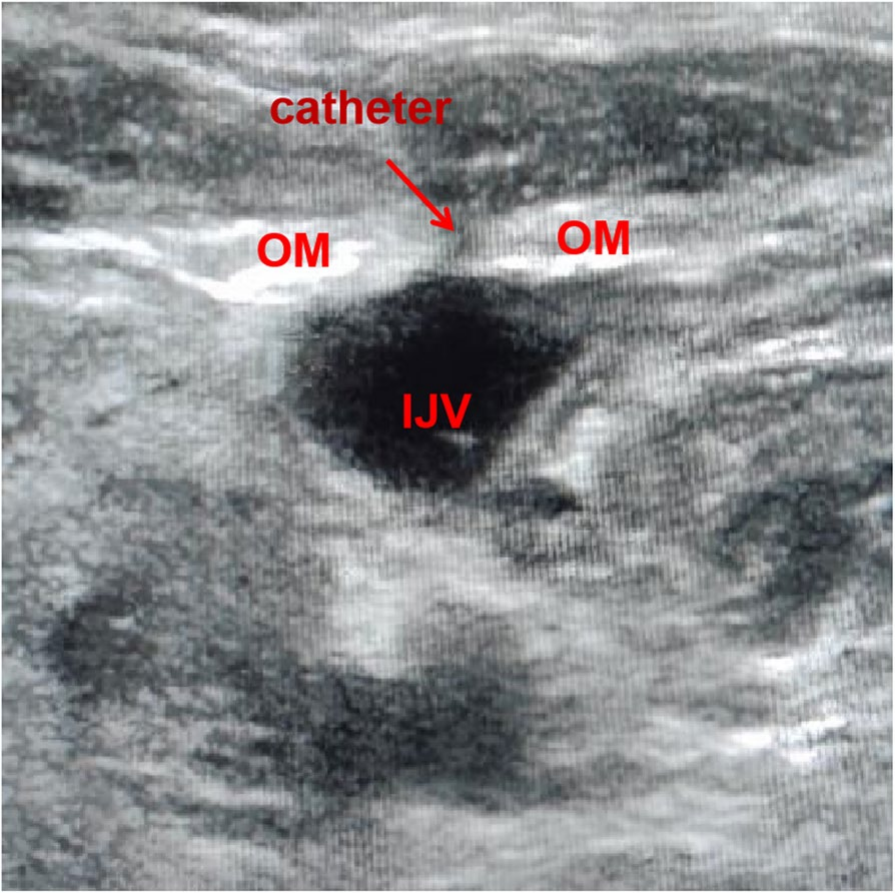
Ultrasound image of catheter traversing the right OMs

We find anaesthesiologists rarely focus on the relationship between the OM and the IJV. But we can still find a close relationship between the two in clinical work (see Fig. 8). In 1988, early literature suggested that the OM was related to the blood flow of the IJV [8, 9]. In addition, we have observed in clinical work that in some cases, obvious neck discomfort (including the pharynx and shoulder) will appear after IJV catheterization. In some cases, it is more apparent when swallowing. Related literature suggests that dysphagia may be related to damage or abnormality of OM[7, 10]. After ultrasonic examination, and it was suspected that the discomfort might be related to the catheter penetrating and connecting the OM during IJV catherization (see Fig. 9).

The present study had some limitations. We only evaluated the anatomic data of healthy volunteers who had never undergone IJV cannulation. And the number of subjects in our study was small. Moreover, we thought that the discomfort in the throat and scapular region might be caused by the injury of the OM during puncture, but no study has confirmed the inevitable relationship between the two. Therefore, further research is needed to confirm our findings. More other studies are also possible, including data from healthy volunteers and patients with different conditions.

The levels of PAST and PMST correspond to the puncture planes of the traditional middle method and posterior method for IJV puncture. Based on the results of this study, it can be seen that the traditional middle approach, if the insertion point plane is too low or the traditional posterior approach is chosen, is more likely to damage the OM and cause discomfort to the patients. At present, in many hospitals, it is impossible to perform IJV catheterization under ultrasound guidance. The traditional middle route puncture point was selected at the top of the triangle, where the sternal head at the lower end of the sternocleidomastoid muscle and the clavicle meet, which could effectively avoid injury to the OM, to an extent. Even ultrasound-guided catheterization of the IJV can cause damage to the OM if it is not well recognised. Therefore, it is crucial for clinicians to popularize the anatomical relationship between the OM and the IJV.

## Conclusions

In conclusion, the traditional middle route puncture point is selected at the top of the triangle where the sternal head at the lower end of the sternocleidomastoid muscle and the clavicle meet, which can effectively avoid injury to the OM, to an extent. Different head rotation angles did not effectively prevent damage to the OM during puncture.

## Data Availability

The data sets generated and /or analysed during the current study are not publicly available due to the manuscript has not been received yet but are available from the corresponding author on reasonable request.

## Abbreviations

PAST: The plane of apex of the sternocleidomastoid triangle
PMST: The plane of middle of the sternocleidomastoid triangle
IJV: Internal jugular vein
OM: Omohyoid muscle
WIJV: Width of the internal jugular vein
WOM: Width of the omohyoid muscle
OWOI: Overlapping width of the omohyoid muscle and internal jugular vein
OR: Overlapping rate

## References

[1] Frykholm P, et al. Clinical guidelines on central venous catheterisation. Acta Anaesthesiol Scand. 2014;58 10.1111/aas.12295 24593804

[2] Malin BR, et al. Mechanical complications of central venous catheter insertions: A retrospective multicenter study of incidence and risks*. *Acta Anaesthesiologica Scandinavica. 2019;63(1):61–68. 10.1111/aas.13214 29992634

[3] Uluer MS, Sargin M, Basaran B (2019). Comparison of the effect of the right lateral tilt position and trendelenburg position on the right internal jugular vein in healthy volunteers: a prospective observational study. J Vasc Access.

[4] Miki I (2014). Anatomical relationship between the common carotid artery and the internal jugular vein during head rotation. Ultrasound.

[5] Mahan AF, McEvoy MD, Gravenstein N (2016). Long-axis view for ultrasound-guided central venous catheter placement via the internal jugular vein. Rom J Anaesth Intensive Care.

[6] Iwanaga J (2017). Unusual muscle of the anterior neck: cadaveric findings with surgical applications. Anatomy & cell biology.

[7] Liu Y (2020). Ultrasound imaging of omohyoid muscle and clinical application value. Journal of Imaging Research and Medical Applications.

[8] Patra P (1988). Physiologic variations of the internal jugular vein surface, role of the omohyoid muscle, a preliminary echographic study. Surg Radiol Anat.

[9] Gianesini S (2014). The omohyoid muscle entrapment of the internal jugular vein. A still unclear pathogenetic mechanism Phlebology.

[10] Ong J, Tham A, Tan J (2021). A systematic review of the omohyoid muscle syndrome (OMS): clinical presentation, diagnosis, and treatment options. Ann Otol Rhinol Laryngol.

